# Pathological and genetic characterization of foot and mouth disease viruses collected from cattle and water buffalo in Egypt

**DOI:** 10.1371/journal.pone.0291970

**Published:** 2023-10-11

**Authors:** Hoda A. Abd-Ellatieff, Asmaa A. Hegazy, Abdel-Rahman A. AbouRawash, Hossam G. Tohamy, Mohammed Al-Shehri, Eman K. Bazh, Hesham Hassan, Bothaina H. Essa

**Affiliations:** 1 Department of Pathology, Faculty of Veterinary Medicine, Damanhour University, Abadiyyat Damanhur, El-Beheira, Egypt; 2 Department of Pathology, Faculty of Veterinary Medicine, Alexandria University, Alexandria, Egypt; 3 Department of Biology, Faculty of Science, King Khalid University, Abha, Saudi Arabia; 4 Department of Parasitology, Faculty of Veterinary Medicine, Menoufia University, Shebin Al-Kom, Egypt; 5 Department of Pathology, College of Medicine, King Khalid University, Abha, Saudi Arabia; 6 Department of Animal Husbandry and Animal Wealth Development, Faculty of Veterinary Medicine, Damanhour University, Abadiyyat Damanhur, El-Beheira, Egypt; Sudan University of Science and Technology, College of Veterinary Medicine, SUDAN

## Abstract

Foot-and-mouth disease (FMD), a highly contagious viral disease caused by FMD virus (FMDV) that threatens Egypt’s livestock industry. FMDV causes severe economic losses in the livestock, with restriction of international trade from endemic regions. Surveillance for FMDV serotypes circulating in Egypt is urgently needed to assess the epidemiological situation in the country. FMD outbreaks reported in Egypt in between December 2016 and January-March 2017. A cross-sectional study was conducted to identify the FMDV serotypes responsible for the outbreaks and to collect information on the virus’s morphopathological effects. Postmortem tissue and clinical samples (oral swabs, vesicular fluids from ruptured vesicles, and blood) were collected from recently deceased and infected animals. Pathological examination revealed classical FMD lesions as vesicular and erosive lesions on epithelial tissues with non-suppurative lymphoplasmacytic myocarditis. Phylogenetic and sequencing analyses demonstrated that FMDV serotype O, EA-3 topotype, VP1 is the prevalent serotype responsible for the pathological alterations and the high mortality in young calves, adult cattle, and water buffalo. The outcomes indicate continuous mutations in the circulating FMDV, which result in the occasional failure of vaccination. Based on these findings, extensive continuous monitoring and serotyping of the existing circulating FMDV isolates and regular vaccination with reevaluation of the currently used vaccine in Egypt are recommended to prevent the recurrence of such outbreaks.

## 1. Introduction

FMD is an acute, highly contagious transboundary disease of domestic and wild cloven-hoofed animals, resulting in severe economic damage to livestock industries [1–3]. FMDV outbreaks have been reported recently in many worldwide areas, such as Nigeria [4], Egypt [5, 6], Ethiopia [7], and other FMD-free countries, including the United Kingdom [8]. FMD is an enzootic disease, and FMDV belongs to the *aphthovirus* genus, Picornaviradae family, and has seven serotypes (A, O C, Asia‐1 and South African Territories (SAT) 1, SAT2, SAT3), with distinct genetic, antigenic and immunologic features [9]. FMD genome is linear, non-segmented single-stranded RNA with approximately 8500 nucleotide bases long, surrounded by an icosahedral capsid consisting of four structural proteins VP1, VP2, VP3, and VP4 [4, 7, 10]. Due to the presence of serotype-specific amino acids, VP1 nucleotide sequencing aids in the determination of variation, geographical circulation, genetic correlations, and differentiations among the various variables of FMDV serotypes [6, 11–13]. The disease is characterized by a high morbidity and mortality rate in young animals and low mortalities in adults (approximately 5%) [14]. The high mortality rate in young animals due to cardiac degeneration and necrosis is estimated to exceed 50% [14, 15].

Variable clinical signs were seen in the affected animals. Nevertheless, most of these signs were vesicular lesions on the oral cavity, feet, tongue, snout, teats, loss of appetite, fever, lameness, and drop in milk production [3, 16–18]. Several outbreaks occurred in many countries, either the endemic settings (such as Africa, Asia, and South America) or even the places that are free from FMDV (e.g., Korea, Japan, Netherlands, United Kingdom, and France) [19–21]. Recently, during the last few years, Egyptian farm animals have been overwhelmed with FMD outbreaks despite applying the vaccination regime in these farms, leading to higher economic losses seasonally. O, A, and SAT2 were the most frequently reported prevalent serotypes in Egypt [6, 18, 22–24]. Infection or vaccination with a specific serotype does not provide cross-protection against other serotypes, as the above-mentioned seven serotypes have a broad spectrum of antigenically distinct subtypes because of the high mutation rate [25–27]. Therefore, continuous surveillance of the circulating FMDV serotypes is urgently required to identify the most suitable vaccine candidate [28] to control FMD. Hence, the current study aimed to genetically characterize the FMDV strains responsible for the outbreaks during 2016–2017, in addition to genetically correlating the isolated serotype with the recently isolated FMDV strains during the last outbreaks in Egypt. Moreover, documentation of the up-to-date morphopathological pictures resulted in high mortality in the affected cattle and buffalo.

## 2. Materials and methods

### 2.1. Study areas

**Fig 1:**
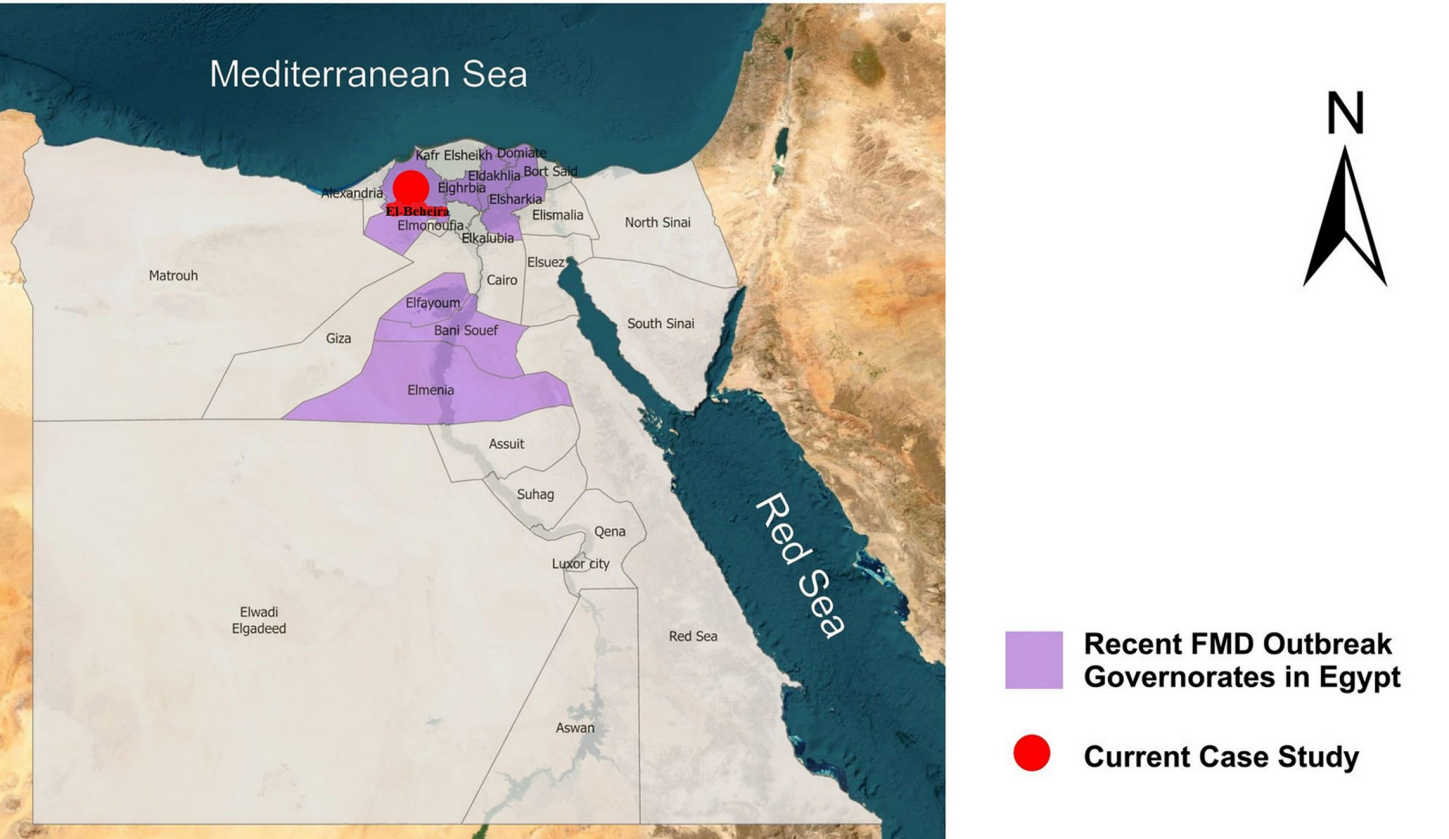
A map showing some recent FMD outbreaks in certain Egyptian governorates.

This study was conducted in El-Beheira provinces in Egypt (Fig 1) where FMDV outbreaks occurred between the end of 2016 and the beginning of 2017. Despite vaccination, veterinarians and owners have observed high mortality rates in calves as well as high mortality rates in cattle and buffalo older than two years. A comprehensive cross-sectional study was conducted to collect samples from a variety of diseased animals suspected to be infected by FMDV. The samples were collected from different clinically infected herds (these herds were FMDV vaccinated). Diseased animals were carefully examined, and the clinical samples (vesicular fluids, blood, serum, and oral swabs) were submitted to the Pathology and Clinical Pathology Lab at the Faculty of Veterinary Medicine, Damanhur University, for diagnosis and investigation.

### 2.2. Animals and clinical signs

The study was performed on five (5) FMDV vaccinated herds (one buffalo, one cattle, three mixed containing cattle and buffalo), as well as eight (8) FMD diseased and dead animals / herd were collected. A total representative of 40 FMD-positive infected animals from five herds were included in that study. Diseased animals displayed severe salivations, depression, loss of appetite, and vesicular lesions in the mouth, tongue, udder, and foot.

### 2.3. Gross pathology and postmortem examination

Postmortem (PM) examinations were conducted immediately on recently deceased animals, according to animal welfare abattoirs and the Research Ethics Committee of Faculty of Veterinary Medicine, Damanhour University, Egypt (DMU-VETMED-PATH-2019-/0145). PM tissues from (the mouth, tongue, foot, rumen, reticulum, omasum, abomasum, liver, lung, intestine, and heart) (n = 170, 4 to 5 PM tissues from the above-mentioned organs/from each dead animal) were collected from recently dead animals during necropsy. Collected samples were washed (2–3 min under tap water), cleaned, labeled, and preserved in a 10% neutral buffered formalin for further histopathological examinations [29].

### 2.4. Samples collection and preservations

Vesicular fluid (from freshly ruptured vesicles and unruptured vesicles) (n = 31) and oral swab samples (n = 18) were collected in specific tubes containing 50% glycerol and 0.04 M phosphate buffer [30]. The collected samples were labeled and kept in ice box containers during transportation to the regional labs at the faculty of Veterinary Medicine, Damanhur University/ Egypt, where they were preserved at -80°C until processing [31]. Conversely, blood samples from suspected animals (5–10 ml blood/ each animal) were collected from the jugular vein on anticoagulant tubes containing EDTA and RNA later and stored at -20°C until RNA extraction.

### 2.5. Virus isolation

Epithelial tissue samples of unruptured and recently ruptured vesicles and oral swab samples were cultured in BHK-21. Cell line(The kindly cell line supplied by Animal Health Research Institute, Virology department, Dokki, Giza). Briefly, tissue samples were ground in sterile sand, pestle, and mortar containing tissue culture medium containing specific antibiotics (penicillin, polymyxin B sulfate, neomycin sulfate, and Mycostatin) [32]. Afterward, the tissue samples homogenate were centrifuged at 2000 rpm for 15 min for clarification and then filtered through membrane filter paper with a pore size of 0.22 μm. Subsequently, the prepared samples were incubated with Confluent monolayer cell cultures, 10% fetal calf serum, and minimum essential medium (MEM). Normal non-infected cells served as control. 0.2–0.4 mL filtered epithelium samples were inoculated to (BHK-21) to investigate the cytopathic effects (CPE) monitored for 24–72 hours according to [7, 31, 33]. Cultured samples that did not show CPE were frozen at ‐ 70°C and reinfected onto BHK-21 cells for a second trial. Positive samples were also examined by the RT-PCR technique.

### 2.6. Histopathology

Fixed specimens in 10% neutral buffered formalin were routinely processed through dehydration in ascending grades of ethanol, cleared in xylene, and embedded in paraffin blocks. Paraffin sections were prepared at 4–5μm thickness on glass slides. The sections were then stained with hematoxylin & eosin and examined using the light microscope [29]. Based on previously described grading systems, the severity of gross and histopathological lesions between calves and adults was evaluated [34–37].

### 2.7. RNA extraction and cDNA synthesis

Total RNA extraction from different samples (vesicular fluid, oral swabs, and blood) was performed using a high pure Qiagen (All Prep® DNA/RNA Mini kit, Germany), according to the manufacturer’s instructions. The extracted samples were then reverse-transcribed according to the manufacturer’s instructions using HiSenScript™ RH (-) cDNA Synthesis kit (NtRONBiotechnology, Korea) according to the manufacturer’s instructions. The obtained cDNA was used for Conventional PCR and RT-PCR using specified primers (Table 1) to amplify FMDV serotypes.

**Table 1:** Sequence, virus specificity, genomic location, and size of PCR amplification product of oligonucleotide primers.

Primer Name	Sequence	Genome direction	Gene	size	Used for
1f	GCC TGG TCT TTC CAG GTC T	+	5’UTR	328	All serotypes detection
1R	CCA GTC CCC TTC TCA GAT C	-	5’UTR		All serotypes
O-1C244F	GCA GCA AAA CAC ATG TCA AAC ACC TT	+	Vp3	1165	Serotype O
O-1C283F	GCC CAG TAC TAC ACA CAG TAC AG	+	Vp3	1124	Serotype O
EUR-2B52R	GAC ATG TCC TCC TGC ATC TGG TTG AT	-	2B		Serotype O/ C/ A/ Asia 1
A-1C562	TACCAAATTACACACGGGAA	+	1c	863	Serotype A
2B208R	ACAGCGGCCATGCACGACAG	-	2B	715	All serotypes
As1–1C530F	CCACRAGTGTGCARGGATGGGT	+	Vp3	886	Serotype Asia 1
As1–1C613F	GCCGGCAARGAYTTTGAGTTYCG	+	Vp3	803	Serotype Asia 1
SAT1–1C559F	GTGTATCAGATCACAGACACACA	+	Vp3	1,043	Serotype SAT 1
SAT2–1C445F	TGGGACACMGGIYTGAACTC	+	Vp3	1,145	Serotype SAT 2
SAT3–1C559F	CTGTACCAAATYACAGACAC	+	Vp3	1,034	Serotype SAT 3
C-1C536	TACAGGGATGGGTCTGTGTGTACC	+	Vp3	883	Serotype C

(+) refers to forward primer; (–) relates to reverse primer

UTR, untranslated region.

1F/1R primer set = Universal primer set for all FMDV serotypes

Any primer set consisted of forward primer (+) and reverse primer (-).

Any primer set consisted of forward primer (+) and reverse primer (-). As (C-1C536/ EUR-2B52R) is a primer set used to detect FMDV serotype (C). (SAT1–1C559F/ 2B208R), is a primer set was used for the detection of FMDV serotype (SAT1).

### 2.8. Conventional PCR analysis

Each cDNA was used as a template for PCR amplification of the 5’UTR of the FMD virus genome using 1F and 1R universal primer for all FMDV serotypes (Table 2), according to [38]. All PCR reaction was carried out using an Eppendorf thermal cycler (SENsQUEsT labcycler). The PCR amplification was performed in a 25 μL volume containing 4 μL DNA, 2 μL dNTP, 1 μL of each primer (10μmol), 2.5μL 10× Ex Taq buffer, 0.25 μL Ex Taq polymerase (Takara, Kyoto, Japan), and 14 μL RNA, DNA free water. The PCR condition for 1F and 1R universal primer was 94°C for 5 min, one cycle; 94°C for 1 min, 55°C for 1 min, followed by 35cycles at 72°C for 2 min with a final extension for 7 min, one cycle. Initially, All suspected samples were tested utilizing a universal primer. Subsequently, positive samples were tested again using serotype-specific RT-PCR (Table 2) by amplifying 1D (VP1region), which is the most variable region of the genome among the seven serotypes utilizing serotype-specific primer [12]. The PCR condition for A-1C562/ EUR-2B52R and C-1C536/ EUR-2B52R primer were set to 94°C for 1 min, one cycle; 62°C for 1 min, followed by 35cycles at 72°C for 2 min with final extension for 7 min, one cycle. Slight modifications were applied to PCR conditions for other primer sets for detecting other serotypes. As the PCR condition for O-1C283F/ EUR-2B52, O-1C244F / EUR-2B52R primer sets were 94°C for 30 seconds, one cycle; 3 to 5 cycles at 60°C for 1 min, followed by 35cycles at 72°C for 2 min with a final extension for 7 min, one cycle. All primers used were supplied by Sigma, Aldrich, Japan.

**Table 2:** Description of selected FMDV samples assigned in the GeneBank.

Gene Bank accession number	sample	Species / age	VP1region (1D gene) of Egyptian isolate
LC384395.1	Epithelial tissue	Cattle / 6month	FMD_EGY1_2017
LC384396.1	blood	Cattle / 3years	FMD_EGY2_2017
LC384397.1	blood	Buffalo/ 3month	FMD_EGY3_2017
LC384398.1	Epithelial tissue	Buffalo / 2 years	FMD_EGY4_2017

### 2.9. DNA purification, sequencing and sequence analysis

According to manufacturer instructions, PCR amplicons were purified and prepared for sequencing using Qiagen® gel extraction kits.

The purified PCR product of the VP1 positive sample was sequenced using its gene-specific primer by 3–500 Genetic analysis, AB applied Biosystem, at colors laboratory, Elmaadi, Cairo, Egypt, EG11431.

The obtained nucleotide sequences of FMDV-positive samples were edited using the sequence scanner software program (http://www.appliedbiosystems.com). The edited sequence was computationally compared with other FMDVs for homology and phylogenetic analysis using Mega 6 program software (www.megasoftware.net/). The phylogenetic trees were generated using the neighbor-joining (N-J) tree method, and the liability of internal branches was assessed by 1000 bootstrap replication. The reference sequences of the FMDV VP1 gene were retrieved from the GeneBank database, and their accession numbers are listed in **S1 Table**.

## 3. Results

### 3.1. Clinical signs expressed by FMDV-infected animals

**Fig 2:**
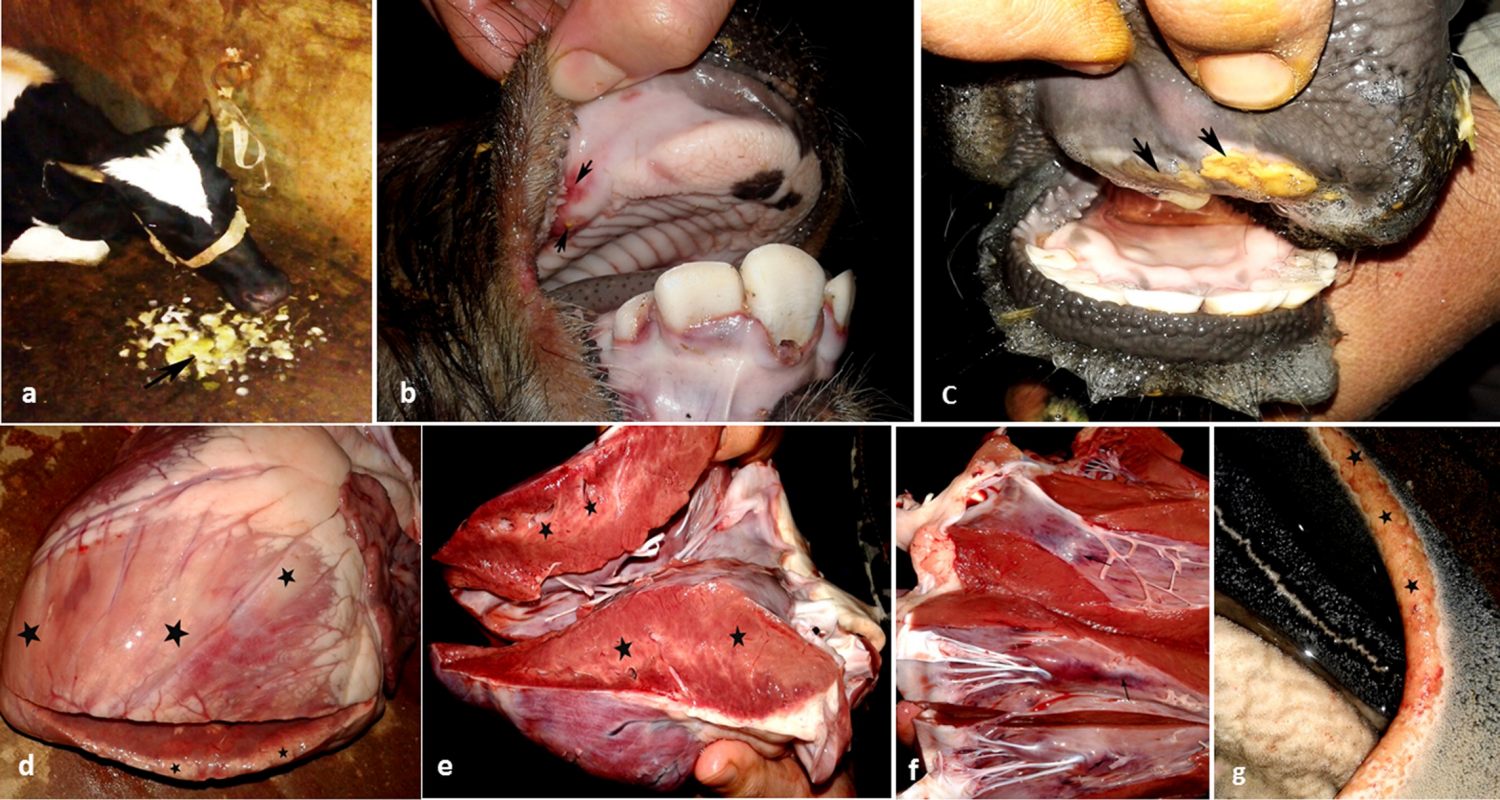
Clinical and gross pathological lesions in calves, cattle and buffalo of FMDV naturally infected animals. **(a)** Heavy salivation of infected cattle. (**b)** Hemorrhagic area of sub mucosa of oral commissure of a calf (arrows). **(c)** Ulcer formation of dental pad with secondary bacterial infection. (**d**) Marked whitish necrosed areas all over the heart (black star). (**e**) A longitudinal section in the heart ventricle and interventricular septum, showing yellowish to whitish streaking of myocardial necrosis (black star)"tiger heart". (**f**) cardial hemorrhage alover the heart atrium, ventricles and papillary muscles (arrows). (**g**) Focal ulcerative lesions of ruminal pillars (arrows).

In cattle, clinical signs varied slightly with age; in adult cattle (over two years), fever ranged from (39°C– 40°C) and persisted for 3–4 days with anorexia. However, in calves, under six months of age, fever reached up to 41°C in some cases, and some animals were suddenly found dead without previous clinical signs. Severe salivation (Fig 2A) with vesicular lesions in the mouth and tongue were seen. Hemorrhage of the oral commissure with subsequently development of ulceration in the upper lips, tongue tips, dental pad, and upper third of the tongue (Fig 2B–2C). In some severely affected cases, the lesions of the foot accompanied by a claw detachment. These lesions were visible on the feet of adult animals older than two years and calves older than six months but not on the feet of animals younger than six months. Animals suffering from severe ulceration of the digits exhibited obvious lameness.

Conversely, clinical signs in buffalo were mild to moderate, mouth lesions were mild in the form of small shallow vesicles with scanty fluid. The foot lesions were absent in calves under six months of age, while the calves aged (6 months to 2 years) and adults (over than two years) showed only mild vesicles and erosion mainly at the bulb of the heal, so lameness was unclear. Prior to death, certain animals exhibited cardiac arrhythmia, followed by dyspnea and grunting.

According to the history of the owners, and veterinarians, the mortality rate was extremely high, particularly among young animals less than six months old, where it reached 100 percent for both cattle and buffalo calves. In addition ahigh fatality rate were recorded also in cattle and buffalo older than six months.

### 3.2. Virus isolation and characterization

Positive epithelial tissues and oral swabs samples collected from calves, cattle, and buffalo during 2016–2017 FMDV outbreaks demonstrated a CPE characterized by swelling, rounding, clumping, granulation, or detachment of monolayer cells from the culture flasks surface, followed by rabid destruction of (BHK-21) cell cultures within 48 to 96 hrs post-inoculation in tissue cultures, indicating the presence of an infectious virus. Samples that did not develop CPE after two passages in cell culture were considered virus-free, and all labeled samples that belonged to these samples were eliminated from the study.

### 3.3. Gross pathology

The mouths and interdigital spaces of the adult cattle and buffalo exhibited numerous blisters, erosions, and ulcerations. Vesicles, irregular-shaped erosions/ulcers of variable size, were typically found on the torus lingua and anterior third of the tongue and more frequently on the gingiva, lip, and dental pad. Hyperemia, ulceration, heel bulb detachment, and sole separation were also identified. Calves exhibited moderate to the severe oral cavity, omasum, abomasum, and rumen erosions and ulcers. As the disease progressed rapidly and the animal died, no foot lesions were detected. All dead calves exhibited myocardial hemorrhage with yellowish-gray streaks in the myocardium (Fig 2D–2F). In addition, similar ulcers were observed in the abomasum and ruminal pillars (Fig 2G). Myocardial hemorrhage (petechial and ecchymotic) with various degrees of myocardial necrosis were also observed.

### 3.4. Histopathological findings

**Fig 3:**
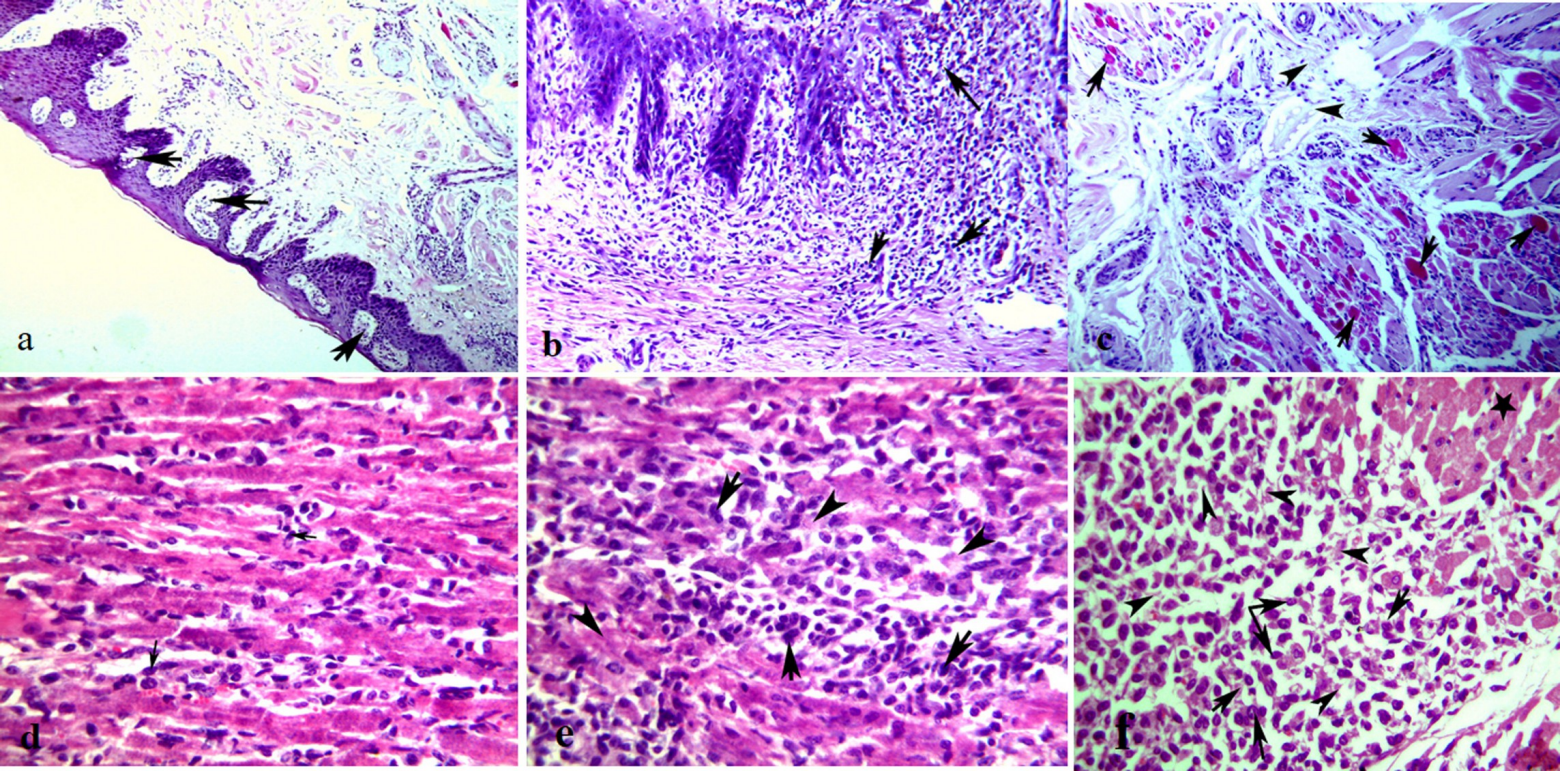
Histopathological lesions in FMDV naturally infected animals. (**a**) Vesicles formations (arrows) with elevation of superficial epithelium in the stratified squamous epithelium of dermis, H&E, X200. (**b**) Vesicular stomatitis of the dental pad of infected calf characterized by leukocytic infiltrations of mucosal and submucosal layer of cornified epithelial tissue (arrows), H&E, X200. (**c**) Zenker^,^s necrosis (arrows) of muscular layers of stratified squamus epithelim with edema inbetween (arrowheads) of cattle, H&E, X200. (**d**) Mild myocarditis of the myocardium of buffalo with mild lymphocytic cell aggregations (arrows), H&E, X400. (**e**) Moderate myocarditis of the myocardium of buffalo calf (less than 2 Y) with moderate lymphocytic cell aggregations (arrows) and mild myocardial muscle necrosis (arrowheads), H&E, X400. (**f**) Severe non- suppurative myocarditis of heart of calf with a significant number of lymphocytic cell aggregations (arrows) with complete lysis of necrosed muscle fibers (arrowheads) and replacement of this muscles by a large number of inflammatory cells (arrows). The black star, referring to the myocardial muscle that still normal not necrosed, H&E, X400.

The histopathological changes of the cornified epithelial tissues of (mouth, tongue, coronary bands, omasum, abomasum, and rumen) were characterized by hydropic degeneration with increased cytoplasmic eosinophilia, micro-abscess formation, hyperkeratosis and subsequent mononuclear cell and granulocyte infiltration in the cells of the stratum spinosum. Intraepithelial bullae and vesicular formations were clearly visible (Fig 3A). Severe lesions exhibited erosions, Zenker^,^s necrosis, and neutrophilic and eosinophilic infiltration of the underlying epithelium (Fig 3B and 3C). Lesions of cornified epithelial tissues (oral cavity and gastrointestinal mucosa (GIT)) were moderate to severe in young calves of cattleand buffalo, adult cattle and buffalo.

The heart was the most severely affected organ and may be the leading cause of death in young calves under six months. Non-suppurative myocarditis, in the form of hyaline degeneration and necrosis of myocytes (hyalinization) with mononuclear cell infiltrations, were the main observed pathological lesions in the heart. Moderate non-suppurative myocarditis was seen in the animals aged six months (Fig 3E). Nevertheless, the calves under six months of age in cattle and buffalo suffered from severe myocarditis and died as a result. In severe cases of myocarditis, necrosed muscle fibers are completely lysed and replaced by a large number of inflammatory cells (Fig 3F). Inflammatory edema with myocardial hemorrhage and vasculitis were also detected. Mild non-suppurative myocarditis (Fig 3D) was not common except in one exceptional case in buffalo aged lower than 2 years and exhpited severe pneumonia.

**Fig 4:**
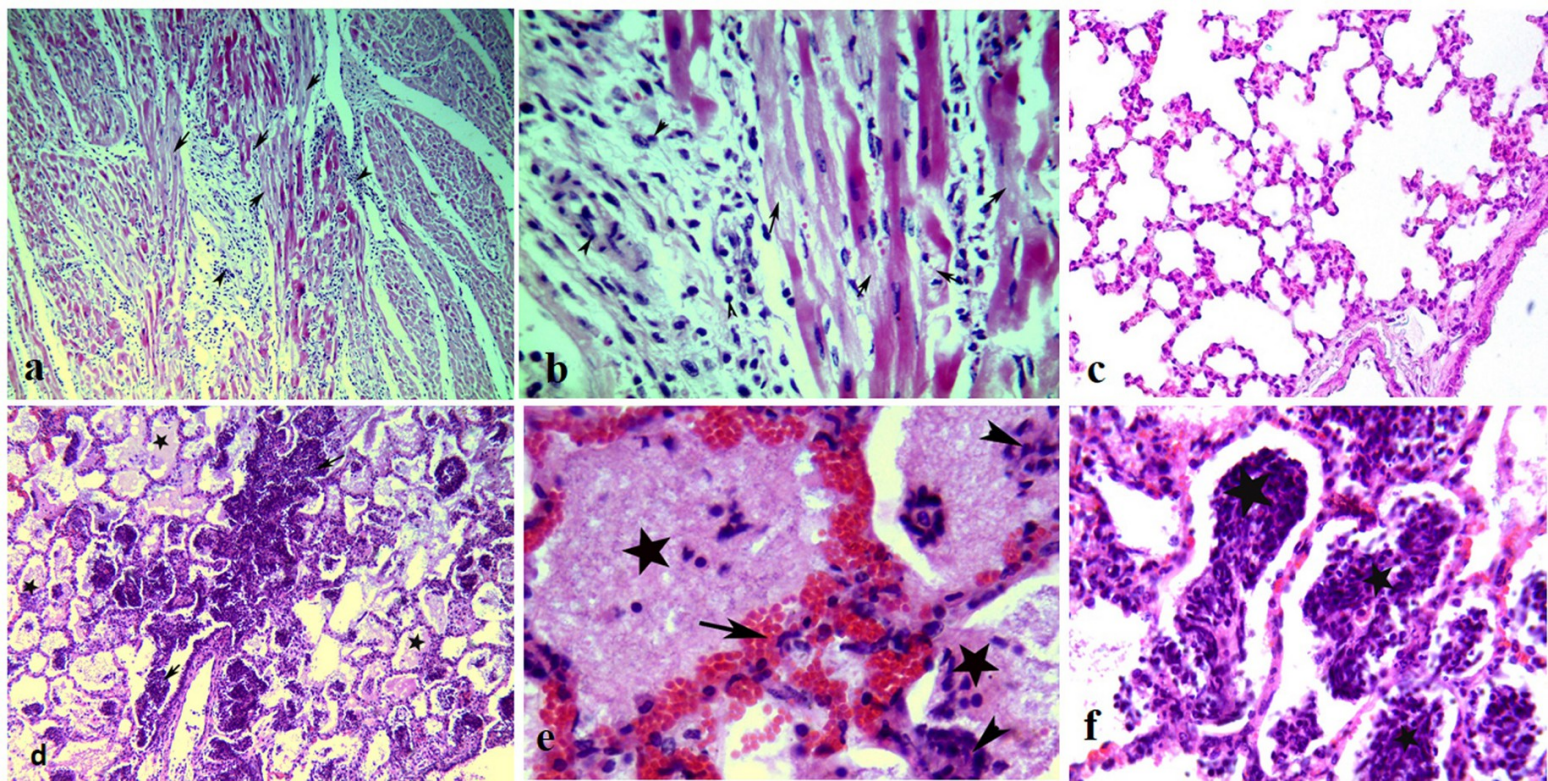
Histopathological lesions of FMDV infected animals. (**a**) Necrosis of myocardial fibers (arrows) of a heart of adult cattle with moderate myocarditis (arrowheads), H&E, X200. (**b**) Degeneration and necrosis of myocardial muscles (arrows) surrounded by inflammatory cells, H&E, X400. (**c)** Normal lung of a buffalo showing normal alveoli, H&E, X100. (**d**) purulent pneumonia of a lung with diffuse infiltration of alveoli by neutrophils and lymphocytic cells (arrows), with serohemorrhagic and fibrinous pneumonia (stars), H&E, X100. (**e**) Congestion of perivascular capillary (arrowheads) by RBCs and leukocytic cells (arrowheads) with serohemorrhagic and fibrinous pneumonia (stars), H&E, X400. (**f**) lung alveoli filled by exudative inflammation (black star) containing significant number of leukocytic cells, H&E, X400.

Hyaline degeneration and coagulative necrosis of myofibers of the muscular layer of the heart (Fig 4A and 4B), tongue, lips, gums, omasum, abomasum, and rumen with focal myositis were also detected.

The lung displayed varying degrees (ranging from mild to severe) of different types of pneumonia (lymphocytic, hemorrhagic, serous, fibrinous, and bronchopneumonia). All that forms of pneumonia were seen alone or mixed with each other’s as serohemorrhagic or serofibrinous (Fig 4D–4F). Moderate to severe lung lesions were seen in adult cattle and calves under two years (except the buffalo calves aged 6 moths-2 years) with mild lesions. However, no lesions were detected in the lung of adult buffalo (Fig 4C).

**Fig 5:**
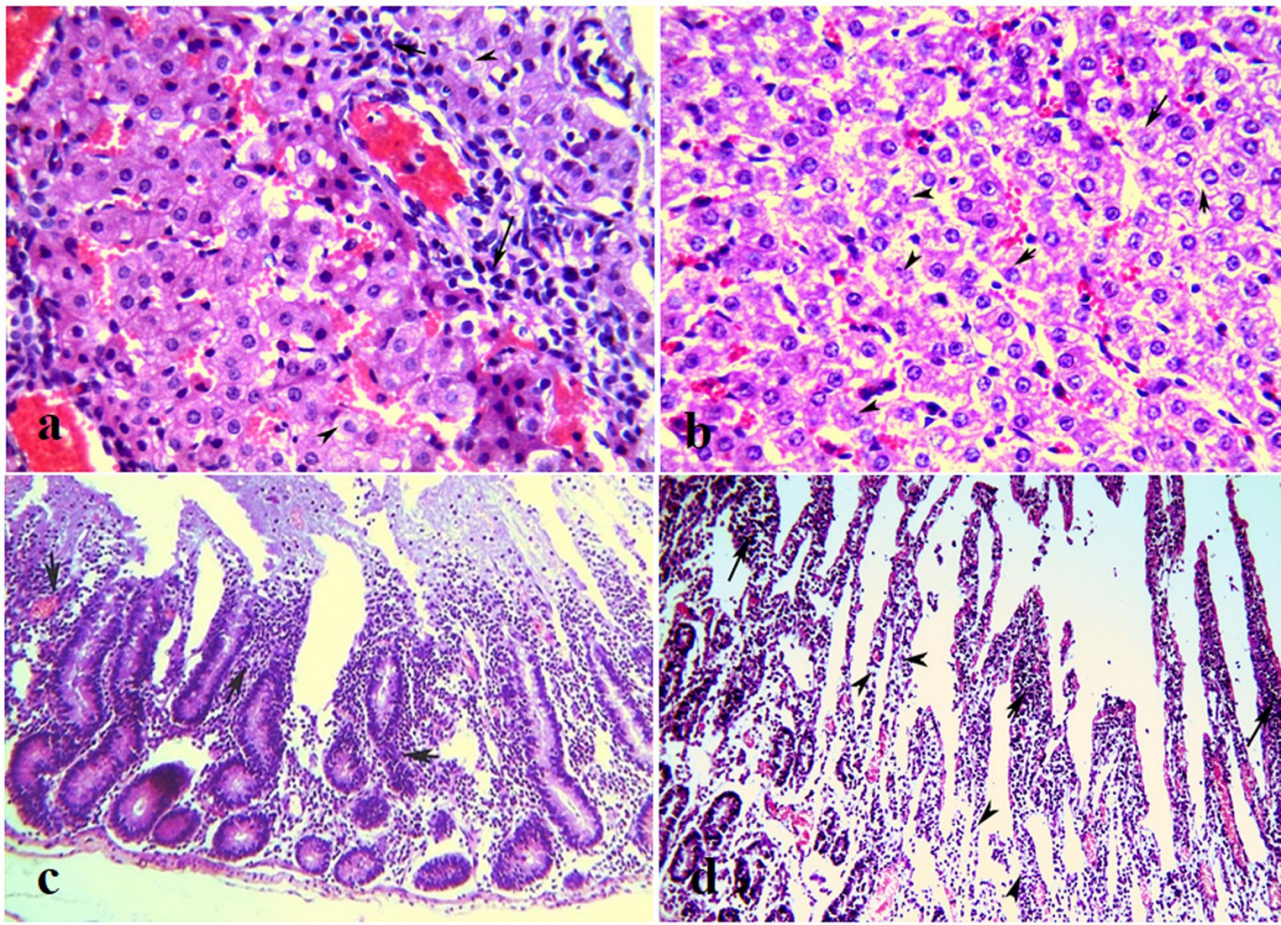
(**a**) liver of buffalo calf, lymphocytic cell aggregations around central hepatic veniules (arrows) with hydropic degeneration (arrowheads) of hepatic cells, H&E, X400. (**b**) Mild hydropic degeneration of hepatic cells (arrows) with mild coagulative necrosis of hepatic cells (arrowheads), H&E, X400. (**c**) The intestine of FMDV infected buffalo showing catarrhal enteritis with leukocytic cell infiltratons of the intestinal villi (arrows), H&E, X100. (**d**) Severe lymphocytic enteritis (arrows) with degeneration of intestinal villi, H&E, X200.

Sporadic cases of examined buffalo and cattle displayed lymphocytic enteritis in the form of leukocytic cell infiltrations of the intestinal villi (Fig 5C and 5D). The liver showed mild to moderate hydropic degeneration of hepatocytes with varying degrees of coagulative necrosis and periportal inflammation (Fig 5A and 5B).

### 3.5. Detection of FMDV by PCR and VP1 region sequencing

Blood and tissue samples extracts were tested using RT-PCR for the presence of viral RNA. Conventional RT-PCR was utilized to detect and serotype FMD in field samples representative of different ages of both species. The PCR results for the universal primer were then examined using specific primers for each serotype. All the results demonstrated that serotype (O) was the responsible serotype for that outbreak. Four samples were sequenced at Elmaadi, laboratory, Cairo, Egypt, EG11431 for genetic characterization of the partial VP1 region, and the nucleotide sequences were submitted to GenBank **(Table 2)** under accession numbers (LC384395, LC384396, LC384397, LC384398).

### 3.6. Nucleotide and amino acid (aa) identities of VP1 region (1D gene) between isolated and another reference FMDV

The nucleotide and its deduced aa sequence alignment analysis of gene encoding for VP1 region were performed between isolated FMDV and 30 references FMDV using the blast sequence analysis program of NCBI (https://blast.ncbi.nlm.nih.gov/Blast.cgi) and MEGA6 program. The identity of the four isolates’ nucleotide sequences ranged from 90% to 96%, with the lowest identity between FMD_EGY1_2017 and FMD_EGY2_2017 and the highest identity between FMD_EGY3_2017 and FMD_EGY4_2017. The identity of the four isolates’ amino acid sequences ranged from 79% to 91%. The highest identity was between FMD_EGY3_2017 and FMD_EGY4_2017 at 91%, and the lowest identity was between FMD_EGY1_2017 and FMD_EGY2_2017 at 79%.

### 3.7. Alignment analysis of nucleotide and deduced amino acid sequences of VP1 region

Alignment analysis of nucleotide and its deduced amino acid sequences of isolated FMDV were performed. The complete genome for FMDV serotype O (A.N: NC_004004.1) was considered a reference strain, and nucleotide mutation, insertion, and deletion were observed. The nucleotide sequence of 4 isolated FMDV showed mutation at position 3242 (C**→**T), 3275 (T**→** G), 3296 (A **→** G), 3300 (G **→** A), 3302 (T **→** C), 3335 (C **→** T), 3338(T **→** G, A, C), 3347 (T **→** C), 3364(A**→**G), 3365 (A**→** C), 3402 (G **→** T), 3437 (A **→** C,T), 3470(G **→**A), 3516 (A**→**G), 3578 (T**→**A, C), 3579 (C **→**T), 3584 (G **→** T,C), 3587(C**→** T), 3596 (A**→** G). Most of these mutation points in 4 isolated FMDV were similar to that of other reference strains of Egypt 2013 and 2014. The nucleotide sequence of FMD_EGY1_2017 showed mutation at positions 3242(G **→**A), 3287 (C **→**A), 3491 (G **→** C), 3512(A **→**G), 3530 (C **→** T). Insertion at two positions 3541 & 3572. The nucleotide sequence of FMD_EGY2_2017 showed insertion at six positions 3541, 3678, 3692, 3701, 3713, and 3731. Nucleotide deletion in FMD_EGY1_2017and, FMD_EGY3_2017 and FMD_EGY4_2017 at position 3692. FMDV serotype O Vaccinal strain used in Egypt **(**EGY/ Sharquia/ 2010, EGY/3/93 vaccine, O1 Manisa), display several points of mutation at positions 3245, 3248, 3260, 3266, 3272, 3290, 3299, 3305, 3311, 3327, 3353,3373,3377, 3380, 3387, 3395, 3401, 3404, 3410, 3416, 3419, 3428,3432, 3434, 3449, 3452, 3456, 3461, 3464, 3482, 3500, 3517,3518, 3521, 3522, 3563, 3590, 3602 (S1 and S2 Figs). These points were somewhat similar to the old FMDV isolated from UKG2001, EGY2006, 2009, and 2010 but it was unidentical with the present study’s FMDV isolates. The amino acid of 4 isolated FMDV displayed mutation at several positions includes 1107(A →G), 1111 (T →A), 1114 (T → A), 1115 (Serine, Glycine → Aspartic Acid), 1116 (Alanine, Proline → Serine), 1127 (Serine, Threonine → Proline), 1131(Cysteine → Arginine). Other points mutation at 1149, 1161, 1172, 1186, 1187, 1233, 1241, 1243, and 1247 were also noticed. FMD_EGY1/2017 display some point mutations at position 1179, 1192, 1227and 1240. Insertion also occurred in FMD_EGY1/2017 at three positions Glutamine at position 1196 by Glycine at position 1206 and Proline at position 1240. FMD_EGY2/2017 showed some insertion points at 1196, 1242, 1251, 1254, 1256, while FMD_EGY4/2017 has one insertion point at 1232 (S3 Fig).

### 3.8. Phylogenetic analysis based on nucleotide and deduced amino acid sequences of VP1 region (1D gene)

**Fig 6:**
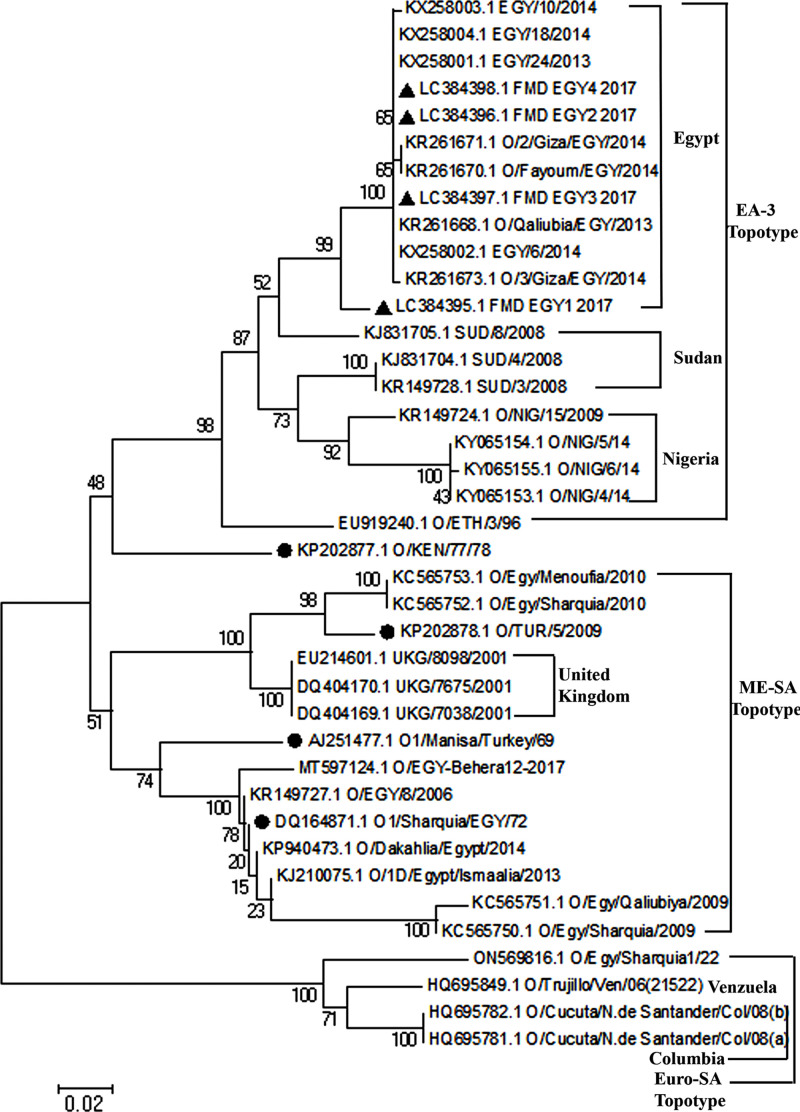
Phylogenetic analysis based on the nucleotide sequence of VP1 gene of Egyptian isolates and other reference strains.

The nucleotide sequences of the VP1 gene of 35 reference strains and four isolated FMDV sequences were analyzed using the MEGA6 program. The Phylogenetic tree showed two clusters. Cluster 1 contain two sub-clusters; the first sub-cluster contains two branches, the first contains four isolated FMDV, O/Qaliubia/EGY/2013, EGY/24/2013, EGY/10/2014, EGY/18/2014, EGY/16/2014, O/2/Giza/EGY/2014, O/3/Giza/EGY/2014, O/Fayoum/EGY/2014 and O/SUD/8/2008, O/SUD/3/2008, O/SUD/4/2008, O/NIG/15/2009, O/NIG/5/14, O/NIG/4/14, O/NIG/6/14, O/NIG/3/14, O/NIG/1/14, O/NIG/9/14, O/NIG/7/14 and O/ETH/3/96, while the second branch contains O/KEN/77/78. The second sub-cluster has two branches, the first contains, Egy/Menoufia/2010, Egy/Sharquia/2010, O/TUR/5/2009 vaccine, UKG/8098/2001, UKG/7675/2001 and UKG/7038/2001, and the second contains O/Manisa/Turkey/69 vaccine, O/EGY-Behera12-2017, O/EGY/8/2006, O1/Sharquia/EGY/72, O/Dakahlia/Egypt/2014, O/1D/Egypt/Ismaalia/2013, Egy/Qaliubiya/2009 and Egy/Sharquia/2009. Cluster 2 contains two sub-clusters; the first contains O/Egy/Sharquia1/22 while the second contains O/Cucuta/N.de Santander/Col/08(a), O/Cucuta/N.de Santander/Col/08(b) and O/Trujuillo/Ven/06(21522) (Fig 6).

## 4. Discussion

FMD has been a highly contagious endemic viral disease affecting cloven-hoofed animals in Egypt since the 1950s [39, 40]. Multiple FMD outbreaks occur annually despite the efforts of the Egyptian government to control and eradicate the virus, and newly emerged lineages of the O, A, and SAT2 serotypes have been identified in Egypt [17, 18, 41]. Despite the mandatory application of locally-produced vaccines containing A, O, and SAT2 lineages, FMD outbreaks continue to recur in Egyptian farms [18, 42–44], with a severe, devastating economic impact on the livestock performance and production in Egypt [17, 41, 44]. The highly contagious nature of the disease and its significant adverse economic impact on the livestock industry motivated us to conduct a comprehensive overview of FMD’s pathological alterations in young calves, adult cattle, and buffalo during 2016–2017 outbreak in Egypt, which resulted in high mortality rates not only in young livestock but also in adults. In addition, we recorded the isolated serotype responsible for that outbreak and compared our results with those of recent studies.

In the current study, vesicle formations at various epithelial sites, including the oral cavity, feet, teats, and ruminal pillars, were observed, followed by erosion and ulcers that often extended during the illness stage. These lesions are similar to the classical form of FMD [18, 45, 46]. Foot lesions ranged from moderate to severe in calves and adult animals. However, no foot lesions were detected in calves aged 1–6 months due to the rapid progression of the disease; the infected calves died prior to the development of foot lesions. According to the owners of the animal herds and veterinarians, the mortality rate in this study was 100% for calves under six months old in both cattle and buffalo, as reported in previous studies [3, 47–49]. This finding was due to viral tropism in the cardiac muscles, resulting in severe myocarditis [41, 47]. Therefore, a high calf mortality rate was anticipated in our study. In contrast to previous FMD studies, which indicated that FMD did not result in high mortality rates in adult animals [15], the high mortality rates in adult buffalo and cattle were unexpected. Buffalo act as reservoirs for specific serotypes; if infected by FMDV, they develop mild or no clinical signs [40, 50]. However, serotypes A and O were under control by the bivalent vaccine (A&O) used in Egypt, as recorded previously [44]. A newly emerging strain belonging to serotype O, the Europe-South America topotype (EURO-SA) lineage, was recorded recently in Egypt and resulted in severe economic losses [18]. Furthermore, the SAT2 serotype resulted in significant mortalities in young and adult cattle and buffalo during the 2012/2013 outbreak in Egypt [22]. This result suggests higher virulence and aggressiveness of the FMDV field circulating serotype, which may be reached Egypt via movements and importation of animals from FMD-infected areas with continuous viral mutations leading to severe histopathological lesions scoring, especially in the hearts and lungs of infected cattle and buffalo.

Heart lesions presented here as myocardial degeneration, Zenkerʼs necrosis, non-suppurative myocarditis, and intense mononuclear cell infiltration in young and infected adult animals. The lesions have varying degrees of severity [51, 52]. Consistent with the myotropic nature of FMDV [47], identical lesions of non-suppurative myositis were also detected in the examined tongue, cheeks, omasum, abomasum, and ruminal muscles. Serofibrinous, serohemorrhagic, and bronchopneumonia were frequently observed, particularly in one-month-to-two-year-old calves. Serofibrinous, serohemorrhagic, and bronchopneumonia were frequently observed, especially in calves aged one month to 2 years [53]. In a few cases, mild-to-moderate hepatic lesions manifested as hepatocellular degeneration and congestion of blood sinusoids, similar to those described by [24, 54]. The virus’s extra-epithelial tropism may be triggered by hepatic degeneration and necrosis.

In our study, conventional RT-PCR was used to detect FMDV using a universal primer [38]. The decrease in the positive rate of the total collected samples may be due to the unstable nature of FMDV RNA and the detection limit of this primer set [55]. Of the seven immunologically different serotypes, A, C, SAT1, 2, 3 (Southern African Territories) and Asia1 [56] circulating worldwide, only serotypes O, A, and SAT2 are prevalent in Egypt [22, 43, 57], while types O, A, SAT1, SAT2, and SAT3 circulate in Africa [58]. In our study, only serotype O, EA-3 topotype was detected using serotype-specific primers. Serotype O is the most prevalent serotype worldwide [30], alone or with other serotypes, and is responsible for several outbreaks in Egypt [18, 42, 59] and other countries, such as Argentina [60], Sri Lanka [61], India [62] and neighboring countries as Palestine, Libya [3, 18, 63] and Sudan [59, 64]. Isolation of FMDV serotype O, EA-3 topotype from Egyptian farms during this FMDV outbreak, however controlling this serotype (O) and serotype A by vaccination in Egypt [44], indicating that the FMDV constitutes an endemic severe health problem for the livestock industry in Egypt [18].

Serotypes are geographically restricted and are described as “topotypes” [65]. These “topotypes” collectively differ in VP1 sequence by at least 15% [66]. Serotype O topotypes found in Africa are EA-1, EA-2, EA-3, EA-4, and MESA (PanAsia-2), from the Middle East. IN EGYPT, serotype O has two topotypes, EA-3 and ME-SA (PanAsia-2) [67]. The present four field isolates of serotype O had been found to belong to the EA-3 topotype, as recorded in other studies [18, 59, 68].

The phylogenetic results obtained from 636 nucleotides of VP1 completely match the obtained when 2208 nucleotides of the complete P1 polyprotein (the genomic region encode all four structural proteins VP1-4) [65]. FMDV serotypes are usually affected by spontaneous mutation points in the VP1 region [69], representing the capsid’s most variable part and containing the leading immunogenic site [70, 71]. Nucleotide sequencing of the VP1 region and phylogenetic analysis has been used extensively to determine the relationships between field isolates; therefore, nucleotide sequencing of the VP1 region and phylogenetic analysis has been used to determine the relationships between our field isolates and other recently isolated Egyptian isolates, and other isolates from some African countries as well as the vaccinal strain used for serotype O in Egypt. The alignment and phylogenetic analyses of the present four isolates revealed that; the new four isolates are closely related to each other by 95% nucleotide identity and to the other reference strains as Egypt 2013 [72], 2014 [73], SUD/8/2008 [74], and Nigeria isolates [75, 76], their identities were 96%, 95%, 91%, 89% respectively. All previously mentioned strains belonged to the EA-3 topotype [67]. In the same scene, the present four isolates were distant from other Egyptian strains 2006, 2009, 2010, 2022, united kingdom strain 2001, and the vaccinal strains used in Egypt (O1 Manisa, pan Asian II (EGY/2010), O/TUR/5/2009, Al-Sharquia 72, O/KEN/77/78), all these strains belong to ME-SA topotype, as previously recorded [59]. The presence of some previously isolated strains within a separate subclade in the phylogenetic tree further enforces our conclusion that the present isolates of FMDV have increased virulence against bovine calves, which could be related to several point mutations in VP1 gene regions of the RNA genome of FMDV [17].

The increased virulence strains among cattle and buffalo in Egypt could be attributed to the introduction of new viral strains through uncontrolled transboundary movements of animals [18, 64, 77]. The sequence and phylogenetic analyses revealed the presence of older isolates and vaccinal strains in separate clusters and subclades far away from the present isolate and recently isolated reference strain, which was grouped in one cluster. Moreover, the presence of sequence divergence between the recent and older isolates and a vaccinal strain far away from the present isolates in the phylogenetic tree could explain the insufficient protection of vaccinated animals against infection with the recent strain. Similar conclusions have been reported by Carrillo [65], who mentioned that the topotype classification system has significant value for vaccine selection.

Finally, we suggest that this increased mortalities in calves, young adults, and adult animals of cattle and buffalo in the last crises of FMD can be attributed to the increased virulence and/or point mutations of the RNA genome circulating strains among animals in Egypt.

Eventually, these findings suggest extensive surveillance and serotyping of the existing field isolates, as well as routine vaccination and reevaluation of the current vaccine in Egypt. Therefore, it is preferable to use vaccines containing the current field strains of FMDV to limit the recurrence of these outbreaks.

## Supporting information

S1 Fig Alignment of the VP1 nucleotide sequence of Egyptian strains and reference strains shows several mutations in new strain and vaccinal strains. (TIF)

S2 Fig Alignment of the VP1 nucleotide sequence of Egyptian strains and reference strains shows several mutations in new strain and vaccinal strains. (TIF)

S3 Fig Alignment of VP1 amino acids sequence of Egyptian strains and reference strains shows several points of mutations in new strain and vaccinal strains. (TIF)

S1 Table FMDV sequences reference strains published in GeneBank. (DOCX)

## References

[1] Grubman MJ, de los Santos T. Aphthovirus. In: Tidona C, Darai G, editors. The Springer Index of Viruses. New York, NY: Springer New York; 2011. p. 1281–6.

[2] Samuel aR, n. J. Knowles Foot-and-mouth disease virus: cause of the recent crisis for the UK livestock industry Trends Genet 2001;17, 421–424. 10.1016/s0168-9525(01)02374-5 11485797

[3] El Damaty HM, Fawzi EM, Neamat-Allah AN, Elsohaby I, Abdallah A, Farag GK, et al. Characterization of foot and mouth disease virus serotype SAT-2 in swamp water buffaloes (Bubalus bubalis) under the Egyptian smallholder production system. Animals. 2021;11(6):1697. doi: 10.3390/ani11061697 34200281 PMC8228956

[4] Ehizibolo DO, De Vleeschauwer AR, Haegeman A, Lefebvre D, Nwosuh CI, Umoh JU, et al. Serological and molecular epidemiology of foot‐and‐mouth disease viruses in agro‐pastoralist livestock herds in the kachia grazing reserve, Nigeria. Transboundary and emerging diseases. 2019;66(4):1575–86. doi: 10.1111/tbed.13182 30901506

[5] Ahmed H, Salem S, Habashi A, Arafa A, Aggour M, Salem G, et al. Emergence of Foot‐and‐Mouth Disease Virus SAT 2 in E gypt During 2012. Transboundary and emerging diseases. 2012;59(6):476–81. doi: 10.1111/tbed.12015 23025522

[6] Hassan AM, Zaher MR, Hassanien RT, Abd-El-Moniem MI, Habashi AR, Ibraheem EM, et al. Molecular detection, phylogenetic analysis and genetic diversity of recently isolated foot-and-mouth disease virus serotype A African topotype, Genotype IV. Virology journal. 2022;19(1):1–9. 34980196 10.1186/s12985-021-01693-y PMC8722054

[7] Sulayeman M, Dawo F, Mammo B, Gizaw D, Shegu D. Isolation, molecular characterization and sero-prevalence study of foot-and-mouth disease virus circulating in central Ethiopia. BMC veterinary research. 2018;14(1):1–10. 29587741 10.1186/s12917-018-1429-9 PMC5870258

[8] Knowles N, Samuel A, Davies P, Kitching R, Donaldson A. Outbreak of foot-and-mouth disease virus serotype O in the UK caused by a pandemic strain. The Veterinary Record. 2001;148(9):258–9. 11292084

[9] Knowles N, Samuel A. Molecular epidemiology of foot-and-mouth disease virus. Virus research. 2003;91(1):65–80. doi: 10.1016/s0168-1702(02)00260-5 12527438

[10] Mason P, Pacheco J, Zhao Q-Z, Knowles N. Comparisons of the complete genomes of Asian, African and European isolates of a recent foot-and-mouth disease virus type O pandemic strain (PanAsia). Journal of general virology. 2003;84(6):1583–93. doi: 10.1099/vir.0.18669-0 12771429

[11] Cottam EM, Haydon DT, Paton DJ, Gloster J, Wilesmith JW, Ferris NP, et al. Molecular epidemiology of the foot-and-mouth disease virus outbreak in the United Kingdom in 2001. Journal of Virology. 2006;80(22):11274–82. doi: 10.1128/JVI.01236-06 16971422 PMC1642183

[12] Knowles NJ, Wadsworth J, Bachanek-Bankowska K, King D. VP1 sequencing protocol for foot and mouth disease virus molecular epidemiology. Rev Sci Tech. 2016;35(3):741–55. doi: 10.20506/rst.35.3.2565 28332654

[13] Carrillo C, Tulman E, Delhon G, Lu Z, Carreno A, Vagnozzi A, et al. Comparative genomics of foot-and-mouth disease virus. Journal of virology. 2005;79(10):6487–504. doi: 10.1128/JVI.79.10.6487-6504.2005 15858032 PMC1091679

[14] Barker iK, dreumel a. A. Van, Palmer n. The alimentary system. In: Pathology of Domestic Animals, 4th ed. (Jubb K. V. F., Kennedy P. C, Palmer N, Eds. Academic Press, San Diego, CA. 1993 pp.141–144.

[15] Alexandersen s. zZ, Donaldson a. I, Garland aJM. The pathogenesis and diagnosis of foot-and-mouth disease. J Comp Pathol. 2003;129, 1–36. doi: 10.1016/s0021-9975(03)00041-0 12859905

[16] Donaldson aI r. F. Sellers Foot-and-mouth disease. In: Diseases of Sheep. (Martin W. N., Aitken I. D, Eds) Blackwell Science, Oxford, United Kingdom. 2000;pp. 254–258.

[17] Hagag NM, Hassan AM, Zaher MR, Elnomrosy SM, Shemies OA, Hussein HA, et al. Molecular detection and phylogenetic analysis of newly emerging foot-and-mouth disease virus type A, Lineage EURO-SA in Egypt in 2022. Virus Research. 2023;323:198960. 10.1016/j.virusres.2022.198960 PMC10194312 36209919

[18] Soltan M, Abd‐Eldiam M, Mahmoud M, Hegazy Y, Amal A, Shafek N. Emergence of Foot and mouth disease virus, serotype O, Europe‐South America topotype in Egypt, 2022. Wiley Online Library; 2022. 10.1111/tbed.14612 35679058

[19] Muroga N, Hayama Y., Yamamoto T., Kurogi A., Tsuda T., Tsutsui T. The 2010 foot-and-mouth disease epidemic in Japan J Vet Med Sci 2012 74, 399–404. 10.1292/jvms.11-0271 22075710

[20] Gibbens JC C. E. Sharpe J. W. Wilesmith L. M. Mansley E. Michalopoulou J. B. Ryan M, et al. Descriptive epidemiology of the 2001 foot-and-mouth disease epidemic in Great Britain: the first five months Vet Rec. 2001;149:729–743. 11808655

[21] Gibbens JC, and Wilesmith J. W. Temporal and geographical distribution of cases of foot-and-mouth disease during the early weeks of the 2001 epidemic in Great Britain Vet Rec 2002;151:407–412. 12403328 10.1136/vr.151.14.407

[22] Elhaig MM, Elsheery MN. Molecular investigation of foot-and-mouth disease virus in domestic bovids from Gharbia, Egypt. Tropical animal health and production. 2014;46(8):1455–62. doi: 10.1007/s11250-014-0665-7 25187028

[23] EL-Bayoumy MK, Abdelrahman KA, Allam A.M, Farag T.K, Abou-Zeina A.A. H, et al. Molecular Characterization of Foot-and-Mouth Disease Virus Collected from Al-Fayoum and Beni- Suef Governorates in Egypt. Global Veterinaria 2014;13(5):828–35.

[24] Abd El Moneim AA, Hafez MH, Ahmed B, Hasanin SA, Algabri N. Pathological and Molecular Investigations on Foot and Mouth Virus Outbreaks Among Cattle Herds in Dakahlia Governorate, Egypt. Zagazig Veterinary Journal (Zag Vet J). 2016;44(2).

[25] Brooksby J. Portraits of viruses: foot-and-mouth disease virus. Intervirology. 1982;18(1–2):1–23. doi: 10.1159/000149299 6288615

[26] Mumford J. Vaccines and viral antigenic diversity. Revue scientifique et technique (International Office of Epizootics). 2007;26(1):69–90. 17633294

[27] Reid SM, Grierson SS, Ferris NP, Hutchings GH, Alexandersen S. Evaluation of automated RT-PCR to accelerate the laboratory diagnosis of foot-and-mouth disease virus. Journal of virological methods. 2003;107(2):129–39. doi: 10.1016/s0166-0934(02)00210-0 12505626

[28] Paton D, Valarcher J, Bergmann I, Matlho O, Zakharov V, Palma E, et al. Selection of foot and mouth disease vaccine strains-a review. Revue scientifique et technique-Office international des épizooties. 2005;24(3):981. 16642769

[29] Bancroft JD, Suvarna KS, Layton C. Bancroft’s Theory and Practice of Histological Techniques E-Book: Elsevier Health Sciences; 2012.

[30] Kitching P, Hammond J, Jeggo M, Charleston B, Paton D, Rodriguez L, et al. Global FMD control—Is it an option? Vaccine. 2007;25(30):5660–4. doi: 10.1016/j.vaccine.2006.10.052 17126959

[31] Arzt J, Baxt B, Grubman M, Jackson T, Juleff N, Rhyan J, et al. The pathogenesis of foot‐and‐mouth disease II: viral pathways in swine, small ruminants, and wildlife; myotropism, chronic syndromes, and molecular virus–host interactions. Transboundary and emerging diseases. 2011;58(4):305–26. doi: 10.1111/j.1865-1682.2011.01236.x 21672184

[32] Hedger R. The isolation and characterization of foot-and-mouth disease virus from clinically normal herds of cattle in Botswana. Epidemiology & Infection. 1968;66(1):27–36. doi: 10.1017/s0022172400040912 4296415 PMC2130609

[33] OIE. Foot and mouth disease. Chapter 3.1.8. Terrestrial Animal Health Code 2021. World Organization for Animal Health, Paris, France. https://www. oie. int/ filea dmin/ Home/ eng/ Health_ stand ards/ tahm/3. 01. 08_ FMD.pdf. 2021.

[34] Gibson-Corley KN, Olivier AK, Meyerholz DK. Principles for valid histopathologic scoring in research. Veterinary pathology. 2013;50(6):1007–15. doi: 10.1177/0300985813485099 23558974 PMC3795863

[35] Cheng Z DD, Zhao L, Wang HL, Doherty TM, Bresee C, Frykman PK. Murine model of Hirschsprung-associated enterocolitis. I: Phenotypic characterization with development of a histopathologic grading system. J Pediatr Surg. 2010; 45:475–482. [PubMed: 20223308]. doi: 10.1016/j.jpedsurg.2009.06.009 20223308 PMC4370315

[36] Cross S. Grading and scoring in histopathology. Histopathology. 1998;33(2):99–106. doi: 10.1046/j.1365-2559.1998.00495.x 9762541

[37] Kleiner DE BE, Natta MV, Behling C, Contos MJ, Cummings OW, Ferrell LD, et al. for the Nonalcoholic Steatohepatitis Clinical Research Network. Design and Validation of a Histological Scoring System for Nonalcoholic Fatty Liver Disease. Hepatol. 2005;41:1313–1321. 10.1002/hep.20701 15915461

[38] Reid SM, Ferris NP, Hutchings GH, Samuel AR, Knowles NJ. Primary diagnosis of foot-and-mouth disease by reverse transcription polymerase chain reaction. Journal of virological methods. 2000;89(1–2):167–76. doi: 10.1016/s0166-0934(00)00213-5 10996650

[39] FAO. Food and agriculture organization. History of FMD disease in Egypt. http://wwwfaoorg/ag/againfo/commissions/eufmd/commissions/eufmd-home/reports/archive/33rd-general-session/fmd-in-egypt/en/. 1999.

[40] Vosloo W, Bastos A, Sangare O, Hargreaves S, Thomson G. Review of the status and control of foot and mouth disease in sub-Saharan Africa. Revue Scientifique Et Technique-Office International Des Epizooties. 2002;21(3):437–45. doi: 10.20506/rst.21.3.1349 12523685

[41] Sobhy NM, Bayoumi YH, Mor SK, El-Zahar HI, Goyal SM. Outbreaks of foot and mouth disease in Egypt: Molecular epidemiology, evolution and cardiac biomarkers prognostic significance. International journal of veterinary science and medicine. 2018;6(1):22–30. doi: 10.1016/j.ijvsm.2018.02.001 30255074 PMC6148740

[42] El-Shehawy L, Abu-Elnaga H, Talat A, El-Garf E, Zakria A, Azab A. A nucleotide sequencing of foot-and-mouth disease virus Egyptian strains. The Journal of American Science. 2011;7(7):430–5.

[43] El-Khabaz KAS, Al-Hosary AAT. Detection and identification of Foot and Mouth disease virus serotypes in Assiut governorate, Egypt. Journal of Advanced Veterinary and Animal Research. 2017;4(1):32–8.

[44] Kandeil A, El-Shesheny R, Kayali G, Moatasim Y, Bagato O, Darwish M, et al. Characterization of the recent outbreak of foot-and-mouth disease virus serotype SAT2 in Egypt. Archives of Virology. 2013;158(3):619–27. doi: 10.1007/s00705-012-1529-y 23132412

[45] Arzt J D. A. Gregg A. Clavijo, and Rodriguez L. L,. Optimization of immunohistochemical and fluorescent antibody techniques for localization of Foot-and-mouth disease virus in animal tissues J Vet Diagn Invest. 2009; 21, 779–792. 10.1177/104063870902100604 19901278

[46] Alexandersen SM N. Foot-and-mouth disease: host range and pathogenesis. Curr Top Microbiol Immunol 2005;288, 9–42. doi: 10.1007/3-540-27109-0_2 15648173

[47] Arzt J, Baxt B., Grubman M. J., Jackson T., Juleff N., Rhyan J.,, et al. The pathogenesis of foot-and-mouth disease II: viral pathways in swine, small ruminants, and wildlife; myotropism, chronic syndromes, and molecular virushost interactions. Transbound Emerg Dis 2011;58, 305–326. doi: 10.1111/j.1865-1682.2011.01236.x 21672184

[48] Karapinar T, Dabak DO, Kuloglu T, Bulut H. High cardiac troponin I plasma concentration in a calf with myocarditis. The Canadian Veterinary Journal. 2010;51(4):397. 20592829 PMC2839829

[49] Gulbahar M, Davis W, Guvenc T, Yarim M, Parlak U, Kabak Y. Myocarditis associated with foot-and-mouth disease virus type O in lambs. Veterinary pathology. 2007;44(5):589–99. doi: 10.1354/vp.44-5-589 17846231

[50] Bronsvoort B, Parida S, Handel I, McFarland S, Fleming L, Hamblin P, et al. Serological survey for foot-and-mouth disease virus in wildlife in eastern Africa and estimation of test parameters of a nonstructural protein enzyme-linked immunosorbent assay for buffalo. Clinical and Vaccine Immunology. 2008;15(6):1003–11. doi: 10.1128/CVI.00409-07 18385460 PMC2446625

[51] Gunes v, Erdogan h. M, Citil m, Ozcan k. Assay of cardiac troponins in the diagnosis of myocardial degeneration due to foot-and-mouth disease in a calf. Vet Rec. 2005;156, 714–715. doi: 10.1136/vr.156.22.714 15923556

[52] Karapinar t, Dabak d. O, t kuloglu h. Bulut High cardiac troponin I plasma concentration in a calf with myocarditis Can Vet J. 2010;51, 397–399. 20592829 PMC2839829

[53] Arzt J J. M. Pacheco, and Rodriguez L. L. The early pathogenesis of foot-and-mouth disease in cattle after aerosol inoculation: identification of the nasopharynx as the primary site of infection. Vet Path 2010;47, 1048–1063. 20587691 10.1177/0300985810372509

[54] El-Amir Y, Hussein H, Sayed M, Aamer A. Clinical, biochemical and pathological findings in buffaloes with foot-and-mouth disease. Journal of Veterinary Advances. 2014;4(9):668–76.

[55] Saeed A, Khan QM, Waheed U, Arshad M, Asif M, Farooq M. RT-PCR evaluation for identification and sequence analysis of foot-and-mouth disease serotype O from 2006 to 2007 in Punjab, Pakistan. Comparative immunology, microbiology and infectious diseases. 2011;34(2):95–101. doi: 10.1016/j.cimid.2009.10.004 20031216

[56] Knowles NJ, Davies PR, Henry T, O’Donnell V, Pacheco JM, Mason PW. Emergence in Asia of foot-and-mouth disease viruses with altered host range: characterization of alterations in the 3A protein. Journal of virology. 2001;75(3):1551–6. doi: 10.1128/JVI.75.3.1551-1556.2001 11152528 PMC114061

[57] El-Bayoumy M, Abdelrahman K, Allam A, Farag T, Abou-Zeina HA, Kutkat M. Molecular characterization of foot and mouth disease virus collected from Al Fayoum and Beni-Suef governorates in Egypt. Glob Vet. 2014;13(5):828–35.

[58] Ayelet G, Mahapatra M, Gelaye E, Egziabher BG, Rufeal T, Sahle M, et al. Genetic characterization of foot-and-mouth disease viruses, Ethiopia, 1981–2007. Emerging infectious diseases. 2009;15(9):1409. doi: 10.3201/eid1509.090091 19788808 PMC2819860

[59] Soltan MA, Negmaldin AH, El-Diasty MM, Mansour SM, Elbadry MA, Wilkes RP. Molecular characterization of circulating Foot and mouth disease virus (FMDV) serotype O topotype EA-3 and serotype A (African topotype) genotype IV in Egypt, 2016. Veterinary microbiology. 2017;208:89–93. doi: 10.1016/j.vetmic.2017.07.018 28888656

[60] König G, Blanco C, Knowles NJ, Palma EL, Maradei E, Piccone ME. Phylogenetic analysis of foot-and-mouth disease viruses isolated in Argentina. Virus Genes. 2001;23(2):175–81. doi: 10.1023/a:1011844204945 11724271

[61] Abeyratne S, Amarasekera S, Ranaweera L, Salpadoru T, Thilakarathne S, Knowles N, et al. The phylogenetic analysis of VP1 genomic region in foot-and-mouth disease virus serotype O isolates in Sri Lanka reveals the existence of’Srl-97’, a newly named endemic lineage. PloS one. 2018;13(3):e0194077. doi: 10.1371/journal.pone.0194077 29570746 PMC5865751

[62] Mahapatra M, Yuvaraj S, Madhanmohan M, Subramaniam S, Pattnaik B, Paton D, et al. Antigenic and genetic comparison of foot-and-mouth disease virus serotype O Indian vaccine strain, O/IND/R2/75 against currently circulating viruses. Vaccine. 2015;33(5):693–700. doi: 10.1016/j.vaccine.2014.11.058 25500306 PMC4315132

[63] Valdazo-González B, Knowles NJ, Hammond J, King DP. Genome sequences of SAT 2 foot-and-mouth disease viruses from Egypt and Palestinian Autonomous Territories (Gaza Strip). Am Soc Microbiol; 2012. doi: 10.1128/JVI.01231-12 22843860 PMC3421706

[64] Ahmed NH, Osman NA, Alfouz W, Saeed HM. Serological detection and genetic characterization of foot-and-mouth disease virus from cattle in northern sudan, 2016‑2018. Veterinary and Animal Science. 2021;13:100188. 34307959 10.1016/j.vas.2021.100188 PMC8283133

[65] Carrillo C. Foot and mouth disease virus genome: Citeseer; 2012.

[66] Domingo E, Martínez-Salas E, Sobrino F, de la Torre JC, Portela A, Ortín J, et al. The quasispecies (extremely heterogeneous) nature of viral RNA genome populations: biological relevance—a review. Gene. 1985;40(1):1–8. doi: 10.1016/0378-1119(85)90017-4 3912262

[67] Lloyd-Jones K, Mahapatra M, Upadhyaya S, Paton DJ, Babu A, Hutchings G, et al. Genetic and antigenic characterization of serotype O FMD viruses from East Africa for the selection of suitable vaccine strain. Vaccine. 2017;35(49):6842–9. doi: 10.1016/j.vaccine.2017.10.040 29102329 PMC5722052

[68] Al-Hosary AA, Kandeil A, El-Taweel AN, Nordengrahn A, Merza M, Badra R, et al. Co-infection with different serotypes of FMDV in vaccinated cattle in Southern Egypt. Virus Genes. 2019;55(3):304–13. doi: 10.1007/s11262-019-01645-3 30771081

[69] Haydon D, Samuel A, Knowles N. The generation and persistence of genetic variation in foot-and-mouth disease virus. Preventive Veterinary Medicine. 2001;51(1–2):111–24. doi: 10.1016/s0167-5877(01)00210-0 11530198

[70] Robertson B, Moore D, Grubman M, Kleid D. Identification of an exposed region of the immunogenic capsid polypeptide VP1 on foot-and-mouth disease virus. Journal of virology. 1983;46(1):311–6. doi: 10.1128/JVI.46.1.311-316.1983 6186823 PMC255125

[71] Sangare O. Molecular Epidemiology of Foot-and-mouth-disease Virus in West Africa: University of Pretoria; 2002.

[72] Diab E, Bazid A-HI, Fawzy M, El-Ashmawy WR, Fayed AA, El-Sayed MM. Foot-and-mouth disease outbreaks in Egypt during 2013–2014: Molecular characterization of serotypes A, O, and SAT2. Veterinary World. 2019;12(2):190. doi: 10.14202/vetworld.2019.190-197 31040557 PMC6460869

[73] Sobhy NM, Mor SK, Mohammed ME, Bastawecy IM, Fakhry HM, Youssef CR, et al. Phylogenetic analysis of Egyptian foot and mouth disease virus endemic strains. The Journal of American Science. 2014;10(9):133–8.

[74] Reeve R, Borley DW, Maree FF, Upadhyaya S, Lukhwareni A, Esterhuysen JJ, et al. Tracking the antigenic evolution of foot-and-mouth disease virus. PloS one. 2016;11(7):e0159360. doi: 10.1371/journal.pone.0159360 27448206 PMC4957747

[75] Ularamu H, Ibu J, Wood B, Abenga J, Lazarus D, Wungak Y, et al. Characterization of foot‐and‐mouth disease viruses collected in Nigeria between 2007 and 2014: Evidence for epidemiological links between West and East Africa. Transboundary and emerging diseases. 2017;64(6):1867–76. doi: 10.1111/tbed.12584 27718336

[76] Xu W, Zhang Z, Nfon C, Yang M. Genetic and antigenic relationship of foot–and–mouth disease virus serotype O isolates with the vaccine strain O1/BFS. Vaccine. 2018;36(26):3802–8. doi: 10.1016/j.vaccine.2018.05.045 29776753

[77] Rady AA, Khalil SA, Torky HA. Molecular epidemiology of FMDV in Northern Egypt (2012–214). Alexandria Journal of Veterinary Sciences. 2014;41(1):120–30.

