# Supplementary figures and images for: Pathological and genetic characterization of foot and mouth disease viruses collected from cattle and water buffalo in Egypt
Source: PLoS One. 2023 Oct 11;18(10):e0291970. doi: 10.1371/journal.pone.0291970 (PMC10566709; doi:10.1371/journal.pone.0291970)

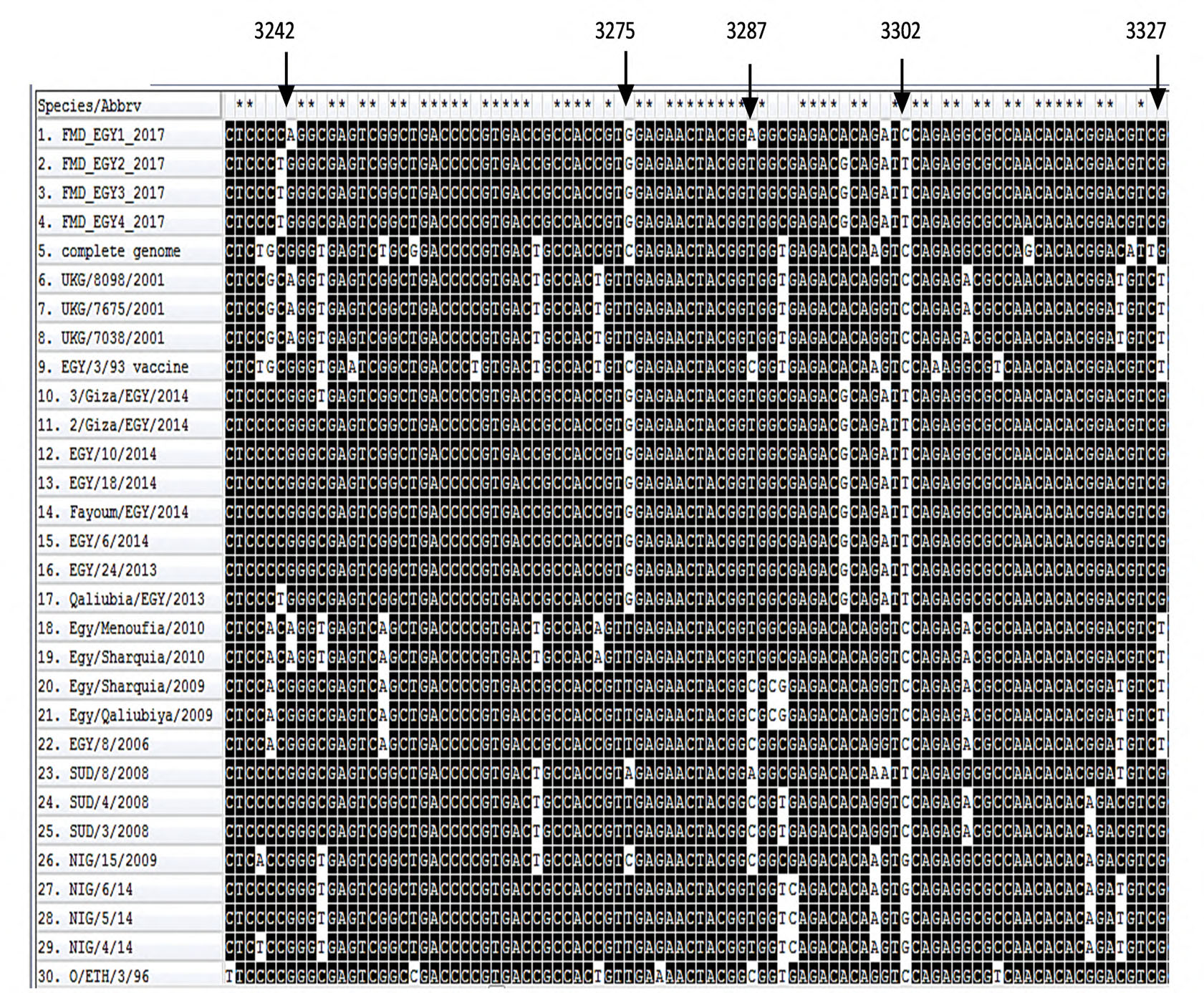

Supplement: S1 Fig — (TIF) [file pone.0291970.s001.tif]

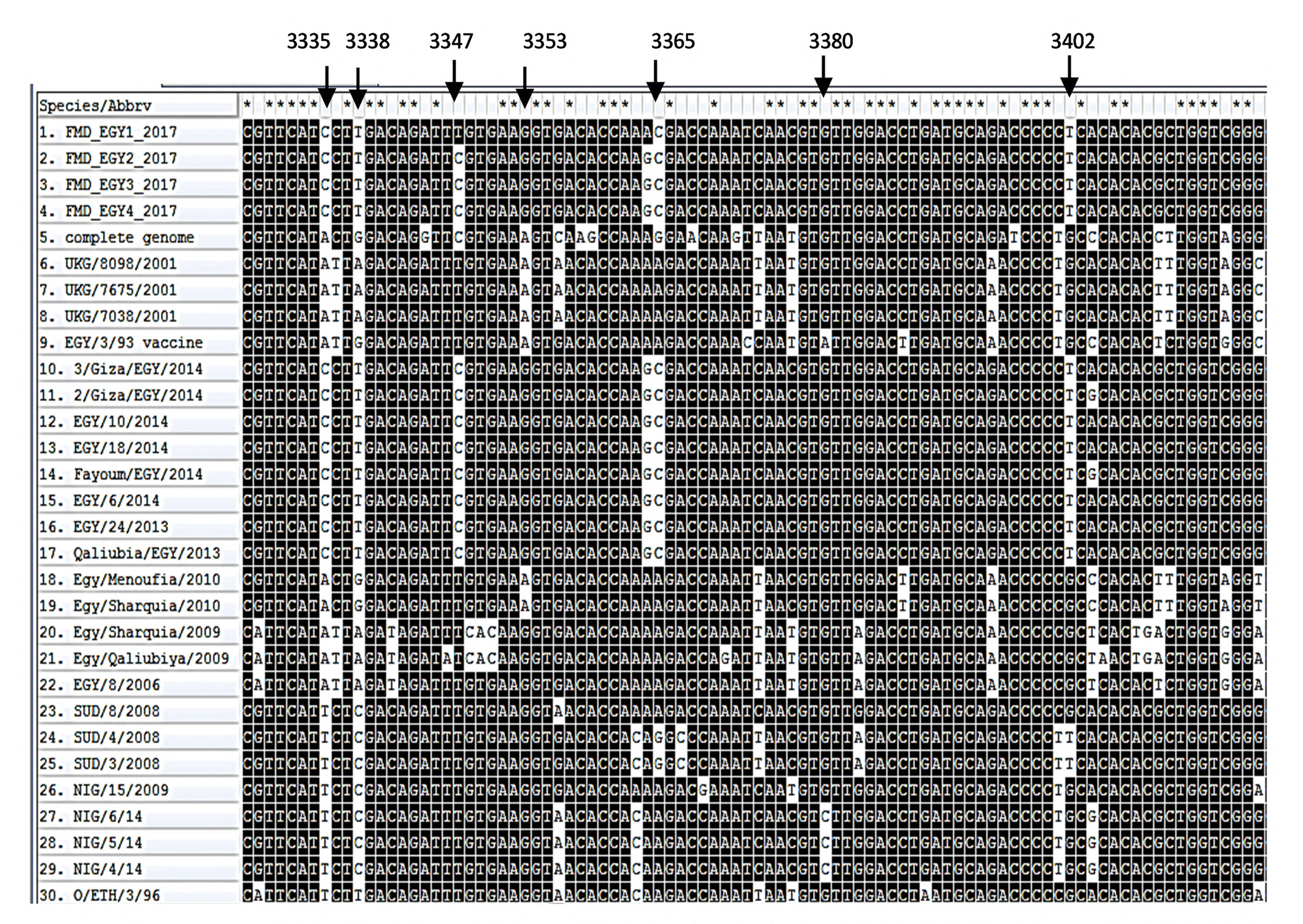

Supplement: S2 Fig — (TIF) [file pone.0291970.s002.tif]

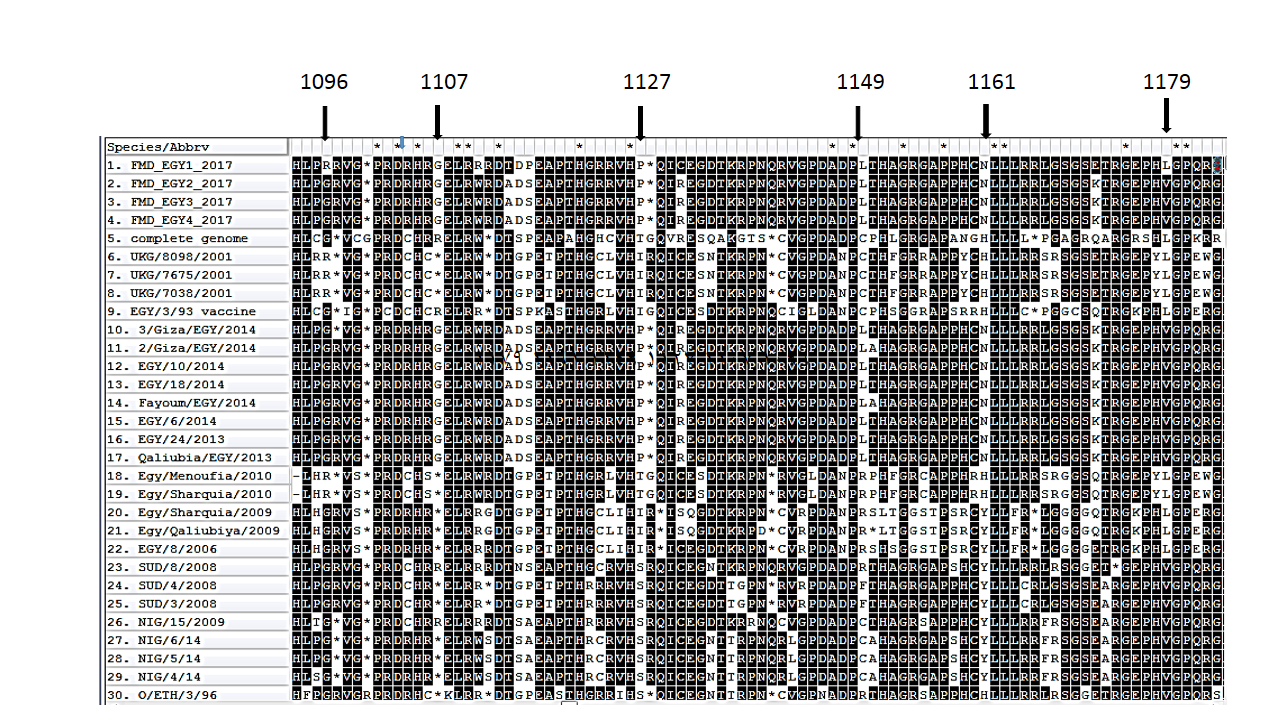

Supplement: S3 Fig — (TIF) [file pone.0291970.s003.tif]
