# Supplementary material for: Pathological and genetic characterization of foot and mouth disease viruses collected from cattle and water buffalo in Egypt
Source: PLoS One. 2023 Oct 11;18(10):e0291970. doi: 10.1371/journal.pone.0291970 (PMC10566709; doi:10.1371/journal.pone.0291970)
Supplement: S1 Table — (DOCX) [file pone.0291970.s004.docx]

**Supplementary Table 1.** Sequences of FMDV reference strains published in GeneBank:

| FMDV strain | topotype | origin | Gene Bank Accession |
| --- | --- | --- | --- |
| SUD/8/2008 | EA-3 | Sudan | KJ831705.1 |
| SUD/4/2008 | EA-3 | Sudan | KJ831704.1 |
| O/SUD/3/2008 | EA-3 | Sudan | KR149728.1 |
| O/NIG/15/2009 | EA-3 | Nigeria | KR149724.1 |
| O/NIG/6/14 | EEA-3 | Nigeria | KY065155.1 |
| O/NIG/5/14 | EA-3 | Nigeria | KY065154.1 |
| O/NIG/4/14 | EA-3 | Nigeria | KY065153.1 |
| O/ETH/3/96 | EA-3 | Ethiopia | EU919240.1 |
| O/Qaliubia/EGY/2013 | EA-3 | Egypt | KR261668.1 |
| EGY/24/2013 | EA-3 | Egypt | KX258001.1 |
| EGY/18/2014 | EA-3 | Egypt | KX258004.1 |
| EGY/10/2014 | EA-3 | Egypt | KX258003.1 |
| EGY/6/2014 | EA-3 | Egypt | KX258002.1 |
| O/3/Giza/EGY/2014 | EA-3 | Egypt | KR261673.1 |
| O/2/Giza/EGY/2014 | EA-3 | Egypt | KR261671.1 |
| O/Fayoum/EGY/2014 | EA-3 | Egypt | KR261670.1 |
| O/Egy/Menoufia/2010 | ME-SA | Egypt | KC565753.1 |
| O/Egy/Sharquia/2010 | ME-SA | Egypt | KC565752.1 |
| O/Egy/Qaliubiya/2009 | ME-SA | Egypt | KC565751.1 |
| O/Egy/Sharquia/2009 | ME-SA | Egypt | KC565750.1 |
| O/EGY/8/2006 | ME-SA | Egypt | KR149727.1 |
| UKG/8098/2001 | ME-SA | United kingdom | EU214601.1 |
| UKG/7675/2001 | ME-SA | United kingdom | DQ404170.1 |
| UKG/7038/2001 | ME-SA | United kingdom | DQ404169.1 |
| O1/Sharquia/EGY/72 | ME-SA | Egypt | DQ164871.1 |
| O/Dakahlia/Egypt/2014 | ME-SA | Egypt | KP940473.1 |
| O/1D/Egypt/Ismaalia/2013 | ME-SA | Egypt | KJ210075.1 |
| O/EGY-Behera12-2017 | ME-SA | Egypt | MT597124.1 |
| O/KEN/77/78 | EA-1 | Kenya | KP202877.1 |
| O/TUR/5/2009 | ME-SA | Turkey | KP202878.1 |
| O/Manisa/Turkey/69 | ME-SA | Turkey | AJ251477.1 |
| O/Egy/Sharquia1/22 | EURO-SA | Egypt | ON569816.1 |
| O/Cucuta/N.de Santander/Col/08(a) | EURO-SA | Columbia | HQ695781.1 |
| O/Cucuta/N.de Santander/Col/08(b) | EURO-SA | Columbia | HQ695782.1 |
| O/Trujuillo/Ven/06(21522) | EURO-SA | Venzuela | HQ695849.1 |
| Beni-suef1/Egy/2017 | NA |  | MF322693.1 |
| A/buffalo/Damietta/ Egypt/2016 | NA |  | MT863268.1 |
| A/cow/Dakahlia/ Egypt/2016 | NA |  | MT863264. 1 |
| Ismailia 2/Egy/2016 | NA |  | KX446997.1 |
| FMDV/SAT2/EGY/Beheria/2019 | SAT2/VII/LIB-12 |  | OK558886 |
| FMDV/SAT2/EGY/Menia/  2018 | SAT2/VII/GHB-12 |  | OK558887 |

East Africa-3 (EA-3) topotype; Middle-East South Asian (ME-SA) topotype; (EURO-SA) Europe-South America topotype; (NA) Not Available.
